# Comparison of Bupivacaine and Lidocaine Use for Postoperative Pain Control in Endodontics

**Published:** 2010-02-20

**Authors:** Saeed Moradi, Neda Naghavi

**Affiliations:** 1. Department of Endodontics, Dental School, Dental Research Center, Mashad University of Medical Sciences, Mashad, Iran

**Keywords:** Bupivacaine, Infiltration, Lidocaine, Maxilla, Pain

## Abstract

**INTRODUCTION:** Many patients suffer from mild, moderate or severe pain during or after root canal therapy. Theoretically, post-operative pain control can be achieved by using long-acting local anesthetics. The aim of this study was to evaluate the efficacy of a long acting anesthesia, bupivacaine, on preventing post-operative pain associated with endodontic treatment, and to compare it with lidocaine.

**MATERIALS AND METHODS:** This study was a double blind and randomized clinical trial on 30 patients' anterior maxillary teeth. The patients were divided into two groups of fifteen. One group was administered lidocanine (2% with 1:100000 epinephrine) local anesthesia and the other group was given bupivacaine (0.5% without epinephrine). The pain in patients were compared using the visual analogue scale (VAS) at definite times *i.e.* before treatment, during treatment and 2,4,6,8,10,12,24,36 and 48 hours after operation. Data were analyzed using One-way ANOVA tests.

**RESULTS:** Bupivacaine significantly decreased postoperative pain compared to lidocaine. Postoperative pain was directly related to preoperative pain. Women reported more pain, though significant difference in postoperative pain report was not found between different ages.

**CONCLUSION:** In conclusion, a single dose of bupivacaine 0.5% used in infiltration anesthesia could be more effective in reduction or prevention of post-operative endodontic pain compared with lidocaine.

## INTRODUCTION

The perceived association of pain with endodontic therapy is a great source of fear for many patients and can prevent them from seeking treatment (1). Controlling post-operative pain represents a meaningful challenge to many practitioners (2). Local anesthetics provides adequate pain relief for the majority of dental treatments, however, failures do occur. These may be the result of anatomical, pharmacological, pharmaceutical, pathological, psychological or technical or iatrogenic factors (3-6).

An objective of endodontic therapy is to relieve and/or prevent patient pain. Good anesthetic technique can considerably eliminate pain during treatments; but, post-treatment endodontic pain remains a significant predicament (7). Post-operative pain control is frequently performed with the administration of short-acting local anesthetic and oral analgesics. Theoretically, pain control can be increased by using a local anesthetic with prolonged action (8-10).

A range of local anesthetic drugs have been used in dentistry. Lidocaine, the first commercialized amide local anesthetic, is still the most widely used anesthetic in some countries (11). It is considered as a reference for new local anesthetics (12). Clinical trials with long-acting anesthetic (bupivacaine and etidocaine) have been performed in patients undergoing oral surgery, endodontic treatment, and periodontal treatment (13-16). Etidocaine was recently withdrawn from the market by Dentsply Pharmaceuticals. Bupivacaine, an amide-type local anesthetic, provides prolonged analgesia and is indicated when post-operative pain is anticipated (17). Its use in routine oral surgery is especially justified for lengthy surgical procedures or oral surgical extraction associated with predicted post-operative pain and discomfort (8,18).

**Table 1:** Studied groups with local anesthetic

**Group**	**n**	**Gender**		**Age (mean)** **Year**
		**Male**	**Female**	
**1** a	**15**	8	7	18-54 (32.4)
**2** b	**15**	9	6	28-61 (41.4)

a *Lidocaine 2% + epinephrine 1:100000*

b *Bupivacaine 2% without epinephrine*

There are many studies on effectiveness and efficient use of bupivacaine for controlling pain after various types of surgery. Gozal *et* *al.* found that using bupivacaine in thyroid surgery significantly decreased post-operative pain and the need for analgesic drugs in these patients (19).

Many studies on patients undergoing surgical removal of impacted third molar showed that bupivacaine significantly decreased post-operative pain and the need for the analgesic drugs compared to short-acting anesthetics (20-22).

Crout *et al. *studied the effectiveness of long-acting anesthesia to reduce pain after periodontal surgery. They concluded that etidocaine postpones the onset of pain and using only lidocaine induced more analgesics use by patients. Also there was no significant difference between etidocaine and bupivacaine in both quality and quantity of anesthesia and post operative pain (14). Fernadez *et al.* compared the amount of pulpal anesthesia obtained with bupivacaine and lidocaine in inferior alveolar nerve blocks. They reported significant anesthetic success with lidocaine for all teeth except the first molars (23). Moore and Dunsky observed no significant difference between lidocaine and bupivacaine in the onset of anesthesia and numbing depth in root canal treatment. The greatest difference between lidocaine and bupivacaine was related to the duration of anesthesia and post-operative discomfort, this is because significantly more patients reported pain in the lidocaine group (2).

Most of these studies investigated the efficacy of bupivacaine in dental and periodontal surgeries. Nevertheless, the anesthetic efficacy of bupivacaine in providing pulpal anesthesia for teeth with irreversible pulpitis and its ability to prevent post-endodontic pain needs further investigation. Therefore, the purpose of this prospective, randomized, double-blind clinical study was to evaluate the efficacy of long acting anesthesia (bupivacaine) to prevent post-operative pain associated with endodontic treatment, and to compare it with lidocaine.

## MATERIALS AND METHODS

The Institutional Ethics Committee of Mashad University of Medical Sciences approved the protocol and the informed consent documents of this study. All patients provided written informed consent before being enrolled. Thirty adult patients (13 women and 17 men), with an age range of 18-61 years participated in this randomized, double-blind clinical trial. They were referred to the Department of Endodontics, Mashad Dental School.

Inclusion criteria were healthy persons (ASA I or II), who required endodontic treatment in upper anterior vital teeth with a clinical diagnosis of irreversible pulpitis and no history of root canal therapy. Exclusion criteria included allergy to amide local anesthetics, pregnancy or lactation, any endocrine or infectious disease, moderate to advanced periodontal disease, nonvital teeth (necrosed).

Bupivacaine was provided from 20 mL Marcaine vials (0.5%, 5mg/mL, Astra co., Sweden) and inserted into the lidocaine cartridges that were emptied previously, and the cartridges were coded by a person who was not directly involved in data collection.

Each patient rated his or her initial pain on a 10-cm Visual Analogue Scale (VAS) with a range of no pain (0 cm) and unbearable pain (10 cm). These 30 patients were randomly divided into two study groups.

Group 1 received 1.8 mL of lidocaine 2% with 1:100.000 epinephrine (Darou Pakhsh co., Tehran, Iran). Group 2 received 1.8 mL of bupivacaine 0.5% without epinephrine (Astra co, Sweden) (Table 1). Topical anesthetic gel *i.e. *lidocaine 2% was passively placed on the infiltration site for 60 seconds using a cotton-tip applicator. Then the two local anesthetic injections were given by using a standard dental aspirating syringe fitted with a 27-gauge, 1.5 inch needle. One operator performed all of the anesthetic procedures. This operator was not involved in data analysis.

**Figure 1:**
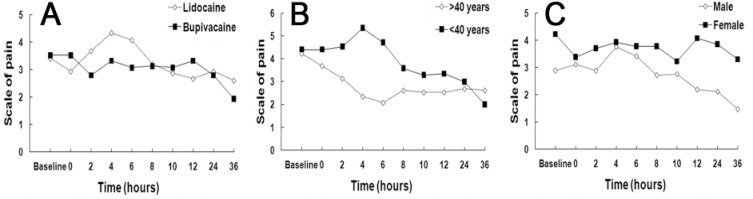
Comparative Visual Analogue Scale in: A) bupivacaine + lidocaine B) patients>40 and <40 years old and C) males and females

The target site was centered over the root apex of the maxillary incisors or canines and anesthetic solution was deposited at the rate of 1 mL/min. After administration of local anesthesia and application of rubber dam, access cavity was prepared. The root canals were instrumented using step-back technique and hand files. Sterile saline solution was used as the irrigant. Then all root canals were filled with gutta-percha by lateral condensation technique by one operator.

Root canal therapy was carried out identically in one visit for all 30 patients. Patients were instructed to rate any occurring pain during the endodontic procedure using VAS. Treatment was deemed successful if no pain or mild discomfort (VAS score of 0 or 1) was felt during access cavity preparation and instrumentation.

A questionnaire was given to the patient at the end of the appointment. Patients were taught how to assess and record the incidence and severity of pain at 2, 4, 6, 8, 10, 12, 24, 36 and 48 hours after the appointment.

Anesthetic success and the degree of post-operative pain among the two groups were analyzed using One-way analysis of variance (ANOVA). Comparisons were also made to evaluate the relation of pain with the other contributing factors such as sex and age with independent t test. Comparisons were considered significant if P was less than 0.05.

## RESULTS

All 30 recruited volunteers completed the trial.

The results of this study were divided into five reports as outlined below.

1- Mean of post-operative pain in bupivacaine and lidocaine groups differed significantly (P<0.05). The mean of post-operative pain in the bupivacaine group was less than the lidocaine group (Figure 1A).

2- Relation of age and pain without considering the kind of anesthetic drug: patients were divided into two groups; less than 40 years (16 patients) and more than 40 years (14 patients). There was no significant difference between these two groups (Figure 1B).

3- Relation of pre- and post-operative pain: without considering the kind of anesthetic drug, there was a significant difference between pre- and post-operative pain (P<0.05).

4- Relation of gender and the amount of pain without considering the kind of anesthetic drug: post-operative pain in women was significantly greater (P<0.01) (Figure 1C).

5- Corresponding impression between gender and time: males and females showed differences in the average of pain at different times after treatment. Maximum pain felt in men was four hours post operation and decreased gradually. However, women experienced maximum pain 12 hours after treatment, and this decreased sharply after 12 hours (Figure 1).

## DISCUSSION

Reduction of post-operative pain is of para-mount importance for patient and dentist. The aim of this study was to evaluate the use of long acting anesthesia for pain control after RCT.

It was hypothesized that long acting anesthetic like bupivacaine would cause effective anesthesia at time of treatment and also would 

be effective in controlling post-operative pain. In the present study the correlation between pre- and post-operative pain is significant and conforms to O'Keefe *et al*. (24). They showed that the probability of post-operative pain in patient with moderate to severe preoperative pain was five times greater than those with a slight pain before treatment. Yesilsoy *et al.* have declared the same conclusion (25).

The effectiveness of bupivacaine in decreasing pain post-operation in endodontics was confirmed by Moore and Dunsky’s study (2). Their research showed that patients treated with lidocaine had significantly more pain after treatment compared with bupivacaine group. Gozal *et al.*'s thyroid surgery study as well as Chapman’s investigation showed decrease of postoperative pain with bupivacaine in surgery, concurring with our study (19,26).

Nespeca *et al*. compared bupivacaine with lidocaine. They found that post-operative pain and use of analgesics were less in the bupivacaine group than the lidocaine group. They also found that there was no significant difference between 0.25% and 0.5% of bupivacaine (27). Studies on third molar surgery showed that bupivacaine significantly decreased post-operative pain and the need for the analgesic drugs compared to short-acting anesthetics (20-22). Crout's *et al.'s *first study, lidocaine was compared with etidocaine and they concluded that etidocaine increased time of no pain; lidocaine group used more analgesics. In the second study, etidocaine was compared to bupivacaine and there was no significant difference between these two groups in both quality and quantity of anesthesia and post operative pain (14).

Our study shows significant difference between male and females in experiencing post-operation pain; women suffered far more pain than men. This concurred with Fox *et al*.'s study (28); however O'Keefe (24), Yesilsoy (25), and Clem's (29) studies did not show any significant difference between the two genders in post-operative pain.

This study concurred with Yesilosoy *et al*.'s similar investigation which did not show any significant difference between the two age groups (25). However, Seltzer (30) and O'Keefe (24) showed differences in >20 years and <20 year age groups; more pain was observed in the over 20 years group. It appears that the difference in conclusions between these two studies and the present study was because of the variation in the age groups.

The reason that bupivacaine is effective in reducing pain is likely to be due to its longer duration of action; this is because it has effective protein binding (23,31).

We must consider that prolonged lip numbness may be disadvantageous and some patients may prefer to manage their pain with analgesics rather than endure lengthy periods of soft tissue anesthesia. However difficulty in eating, speaking, and possibility of soft tissue trauma, occurs more often in block injections than infiltration injections. Also, as the rate of post-operative pain post-endodontic treatment is as follows: 57%, no pain; 21%, mild pain; 15%, moderate pain and 7%, severe pain (32), it may be wise to utilize long-acting analgesic drugs only in patients expecting to have post-operative moderate or severe pain.

## CONCLUSION

Within the limitations of this study (low sample size) we can safely conclude that bupivacaine infiltration injections were more successful than lidocaine infiltration for post-endodontic pain control in maxillary anterior teeth.

Moreover, the severity of pain after treatment was directly related to the pain before treatment.

## References

[1] Ingle JI, Bakland LK, Baumgartner JC, Gibbs JL, Hargreaves KM (2008). Mechanism of odontogenic and non-odontogenic pain. Ingle's Endodontics 6.

[2] Moore PA, Dunsky JL (1983). Bupivacaine anesthesia--a clinical trial for endodontic therapy. Oral Surg Oral Med Oral Pathol.

[3] Byers MR, Taylor PE, Khayat BG, Kimberly CL (1990). Effects of injury and inflammation on pulpal and periapical nerves. J Endod.

[4] Wong MK, Jacobsen PL (1992). Reasons for local anesthesia failures. JAmDentAssoc.

[5] Quinn CL (1998). Injection techniques to anesthetize the difficult tooth. J CalifDentAssoc.

[6] Hargreaves KM, Keiser K (2002). Local anesthetic failure in endodontics. Endodontic Topics.

[7] Marshall JG, Walton RE (1984). The effect of intramuscular injection of steroid on posttreatment endodontic pain. J Endod.

[8] Marković AB, Todorović L (2006). Postoperative analgesia after lower third molar surgery: contribution of the use of long-acting local anesthetics, low-power laser, and diclofenac. Oral Surg Oral Med Oral Pathol Oral Radiol Endod.

[9] Fisher SE, Frame JW, Rout PG, McEntegart DJ (1988). Factors affecting the onset and severity of pain following the surgical removal of unilateral impacted mandibular third molar teeth. Br Dent J.

[10] Gregorio LV, Giglio FP, Sakai VT, Modena KC, Colombini BL, Calvo AM, Sipert CR, Dionísio TJ, Lauris JR, Faria FA, Trindade Junior AS, Santos CF (2008). A comparison of the clinical anesthetic efficacy of 4% articaine and 0.5% bupivacaine (both with 1:200,000 epinephrine) for lower third molar removal. Oral Surg Oral Med Oral Pathol Oral Radiol Endod.

[11] Corbett IP, Ramacciato JC, Groppo FC, Meechan JG (2005). A survey of local anaesthetic use among general dental practitioners in the U.K. attending post-graduate courses on pain control. Br Dent J.

[12] Brunetto PC, Ranali J, Ambrosano GM, de Oliveira PC, Groppo FC, Meechan JG, Volpato MC (2008). Anesthetic efficacy of 3 volumes of lidocaine with epinephrine in maxillary infiltration anesthesia. Anesth Prog.

[13] Davis WM Jr, Oakley J, Smith E (1984). Comparison of the effectiveness of etidocaine and lidocaine as local anesthetic agents during oral surgery. Anesth Prog.

[14] Crout RJ, Koraido G, Moore PA (1990). A clinical trial of long-acting local anesthetics for periodontal surgery. Anesth Prog.

[15] Linden ET, Abrams H, Matheny J, Kaplan AL, Kopczyk RA, Jasper SJ Jr (1986). A comparison of postoperative pain experience following periodontal surgery using two local anesthetic agents. J Periodontol.

[16] Dunsky JL, Moore PA (1984). Long-acting local anesthetics: a comparison of bupivacaine and etidocaine in endodontics. J Endod.

[17] Reader A, Nusstein J, Hargreaves KM, Cohen S, Hargreaves KM (2006). Local anesthesia in endodontics. Pathways of the pulp.

[18] Volpato MC, Ranali J, Ramacciato JC, de Oliveira PC, Ambrosano GM, Groppo FC (2005). Anesthetic efficacy of bupivacaine solutions in inferior alveolar nerve block. Anesth Prog.

[19] Gozal Y, Shapira SC, Gozal D, Magora F (1994). Bupivacaine wound infiltration in thyroid surgery reduces postoperative pain and opioid demand. Acta Anaesthesiol Scand.

[20] Hyrkäs T, Ylipaavalniemi P, Oikarinen VJ, Paakkari I (1994). Effective postoperative pain prevention through administration of bupivacaine and diclofenac. Anesth Prog.

[21] Neal JA, Welch TB, Halliday RW (1993). Analysis of the analgesic efficacy and cost-effective use of long-acting local anesthetics in outpatient third molar surgery. Oral Surg Oral Med Oral Pathol.

[22] Bouloux GF, Punnia-Moorthy A (1999). Bupivacaine versus lidocaine for third molar surgery: a double-blind, randomized, crossover study. J Oral Maxillofac Surg.

[23] Fernandez C, Reader A, Beck M, Nusstein J (2005). A prospective, randomized, double-blind comparison of bupivacaine and lidocaine for inferior alveolar nerve blocks. J Endod.

[24] O'Keefe EM (1976). Pain in endodontic therapy: preliminary study. J Endod.

[25] Yesilsoy C, Koren LZ, Morse DR, Rankow H, Bolanos OR, Furst ML (1988). Post-endodontic obturation pain: a comparative evaluation. Quintessence Int.

[26] Chapman PJ (1988). A controlled comparison of effectiveness of bupivacaine for post-operative pain control. Aust Dent J.

[27] Nespeca JA (1976). Clinical trials with bupivacaine in oral surgery. Oral Surg Oral Med Oral Pathol.

[28] Fox J, Atkinson JS, Dinin AP, Greenfield E, Hechtman E, Reeman CA, Salkind M, Todaro CJ (1970). Incidence of pain following one-visit endodontic treatment. Oral Surg Oral Med Oral Pathol.

[29] Clem WH (1970). Posttreatment endodontic pain. J Am Dent Assoc.

[30] Seltzer S, Bender IB, Ehrenreich J (1961). Incidence and duration of pain following endodontic therapy, Relationship to treatment with sulfonamides and to other factors. Oral Surg Oral Med Oral Pathol.

[31] Ruetsch YA, Böni T, Borgeat A (2001). From cocaine to ropivacaine: the history of local anesthetic drugs. Curr Top Med Chem.

[32] Georgopoulou M, Anastassiadis P, Sykaras S (1986). Pain after chemomechanical preparation. Int Endod J.

